# Melatonin supplementation to treat the metabolic syndrome: a randomized controlled trial

**DOI:** 10.1186/1758-5996-6-124

**Published:** 2014-11-18

**Authors:** Abhinav Goyal, Paul D Terry, Hillary M Superak, Christine L Nell-Dybdahl, Ritam Chowdhury, Lawrence S Phillips, Michael H Kutner

**Affiliations:** Department of Medicine, Division of Cardiology, Emory University School of Medicine, Atlanta, GA USA; Department of Epidemiology, Emory Rollins School of Public Health, Atlanta, GA USA; Departments of Surgery and Public Health, University of Tennessee, 1914 Andy Holt Ave., HPER 390, Knoxville, TN 37996 USA; Department of Biostatistics and Bioinformatics, Emory Rollins School of Public Health, Atlanta, GA USA; Emory Center for Heart Disease Prevention, Emory Healthcare, Atlanta, GA USA; James T. Laney School of Graduate Studies, Emory University, Atlanta, GA USA; Department of Medicine, Division of Endocrinology, Emory University School of Medicine, Atlanta, GA USA

**Keywords:** Melatonin, Metabolic syndrome, Randomized controlled trial

## Abstract

**Background:**

Supplemental melatonin may ameliorate metabolic syndrome (MetS) components, but data from placebo-controlled trials are lacking.

**Methods:**

We conducted a double-blind, placebo-controlled, crossover, Phase II randomized pilot clinical trial to estimate the effects of melatonin supplementation on MetS components and the overall prevalence of MetS. We randomized 39 subjects with MetS to receive 8.0 mg oral melatonin or matching placebo nightly for 10 weeks. After a 6-week washout, subjects received the other treatment for 10 more weeks. We measured waist circumference, triglycerides, HDL cholesterol, fasting glucose, and blood pressure (BP) in each subject at the beginning and end of both 10-week treatment periods. The primary outcome was the mean 10-week change in each MetS component, and a secondary outcome was the proportion of subjects free from MetS, after melatonin versus placebo.

**Results:**

The mean 10-week change for most MetS components favored melatonin over placebo (except fasting glucose): waist circumference -0.9 vs. +1.0 cm (p = 0.15); triglycerides -66.3 vs. -4.2 mg/dL (p = 0.17); HDL cholesterol -0.2 vs. -1.1 mg/dL (p = 0.59); fasting glucose +0.3 vs. -3.1 mg/dL (p = 0.29); systolic BP -2.7 vs. +4.7 mmHg (p = 0.013); and diastolic BP -1.1 vs. +1.1 mmHg (p = 0.24). Freedom from MetS tended to be more common following melatonin versus placebo treatment (after the first 10 weeks, 35.3% vs. 15.0%, p = 0.25; after the second 10 weeks, 45.0% vs. 23.5%, p = 0.30). Melatonin was well-tolerated.

**Conclusions:**

Melatonin supplementation modestly improved most individual MetS components compared with placebo, and tended to increase the proportion of subjects free from MetS after treatment.

**Trial registration:**

NCT01038921, clinicaltrials.gov

## Background

The metabolic syndrome (MetS) is a cluster of metabolic risk factors with increasing worldwide prevalence [1]. The metabolic syndrome has been strongly associated with the risk of adverse cardiovascular events, new-onset diabetes mellitus, and all-cause mortality [2]. Therefore, there is great interest in identifying interventions that can reduce the risk of adverse outcomes in persons with MetS.

Melatonin (N-acetyl-5-methoxytryptamine) is an endogenous indoleamine hormone (chemical structure C_13_H_16_N_2_O_2_) that is synthesized and secreted by the pineal gland. Melatonin release into the circulation is augmented in darkness and decreased during exposure to light, and facilitates the orientation of the body’s physiologic systems according to circadian patterns [3]. Animal studies and non-randomized human studies suggest that melatonin supplementation may ameliorate components of MetS, including elevated glucose and insulin resistance, hypertension, dyslipidemia, and obesity [4–7]. In addition, human genome-wide association studies suggest a link between circadian rhythm regulation and glucose homeostasis mediated through the melatonin signaling pathway [8–10]. Studies also implicate altered plasma melatonin rhythms in the etiology of type 2 diabetes [11] as well as MetS [12]. Epidemiologic studies suggest an inverse relationship between nocturnal melatonin secretion and insulin resistance [13, 14]. However, randomized, placebo-controlled trials in humans are lacking.

We conducted a pilot randomized, double-blind, crossover, placebo-controlled, Phase II clinical trial of melatonin supplementation in 39 women and men with MetS to determine whether melatonin is efficacious in ameliorating the individual MetS components (plasma glucose, blood pressure, triglycerides, waist circumference, and HDL-cholesterol), and in increasing the proportion of subjects free from MetS after treatment.

## Methods

The design and rationale of this trial has been published [15]. Screening of the first patient occurred in April 2010, enrollment ended December 2011, and trial follow-up was completed July 2012. The Institutional Review Board of Emory University approved the study. This report conforms with the CONSORT standards for reporting of randomized controlled trials [16].

### Entry criteria

Eligible subjects met three or more of the following five MetS criteria established by the National Heart, Lung, and Blood Institute and the American Heart Association [17]: elevated fasting glucose (≥100 mg/dL), elevated blood pressure (>130 mmHg systolic blood pressure [SBP] or >85 mmHg diastolic diastolic blood pressure [DBP]), elevated triglycerides (≥150 mg/dL), reduced HDL cholesterol (<40 mg/dL in men, <50 mg/dL in women), and enlarged waist circumference (≥102 cm in men, ≥88 cm in women). Subjects had to meet these criteria even if on risk factor treatment. We excluded subjects with diabetes because we were interested in the effect of melatonin on MetS before diabetes had developed. We excluded subjects taking calcium channel blockers based on data that melatonin may interact with calcium channel blockers to increase blood pressure [18].

### Recruitment, screening, and randomization

Participants were recruited from sources within Emory Healthcare, including subjects with MetS from a prior study funded by the National Institute of Diabetes and Digestive Kidney; the Preventive Cardiology Clinic at The Emory Clinic in Atlanta, GA; and advertisements posted throughout The Emory Clinic. Screening included a detailed chart review, in-person interviews, physical exam, blood tests to document the presence of MetS, and oral glucose tolerance testing to rule out diabetes. Individuals meeting entry criteria entered a 10-day run-in phase to assess adherence to therapy. Those who took at least 80% of their run-in tablets without reporting intolerable side effects were randomized.

**Figure 1:**
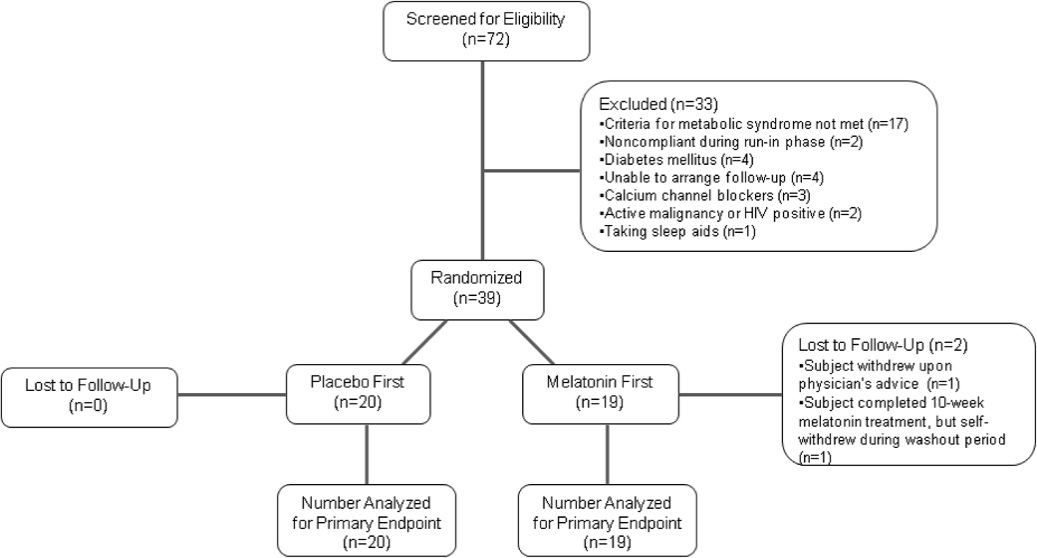
**Study flow diagram.**

**Figure 2:**
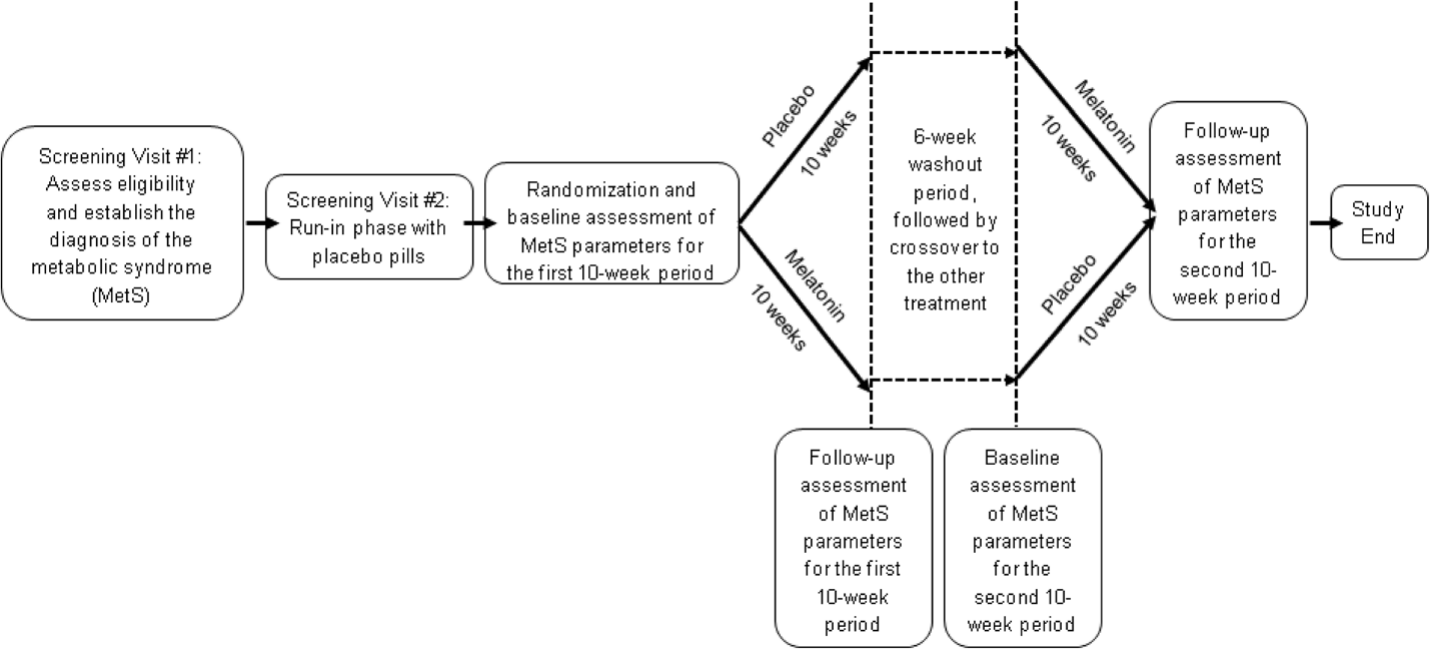
**Crossover study design and measurement intervals.**

A total of 72 subjects were screened for eligibility (Figure 1), of which 33 were excluded during screening and run-in phases. For the 39 randomized subjects, an independent biostatistician randomly generated four permuted blocks of size 10 (five melatonin and five placebo), with which subjects were randomized to receive either 8.0 mg oral melatonin or matching placebo daily (one hour before bedtime) for 10 weeks. After the first 10 weeks, each subject underwent a 6-week washout period, and then crossed over to receive the alternative treatment for another 10-weeks (Figure 2). Study investigators, participants and their personal health care providers, and laboratory staff were blinded to treatment assignment.

### Melatonin treatment

As previously described [15], we studied a daily dose of 8.0 mg melatonin compared with placebo to be taken one hour before bedtime. The Investigational Drug Service at Emory University purchased melatonin in powder form, compounded the melatonin into 8.0 mg immediate-release melatonin in gelatinous capsules, and also made the matching placebo capsules. A daily dose of 8.0 mg of immediate-release melatonin was chosen based on prior studies showing similar side effect profiles between melatonin doses as high as 7–8 mg and placebo [19]. The duration of 10 weeks of study treatment for each of the melatonin and placebo treatment phases was based on human studies that showed that two months of melatonin therapy effected important changes in metabolic parameters including blood pressure, glucose, and serum lipids [7].

### Measurements

We first measured the five MetS components at screening to determine study eligibility. Following randomization, we again measured the five MetS components in each enrolled subject at four additional time points (Figure 2): 1) at the beginning of the first 10-week period of melatonin or placebo administration; 2) at the end of the first 10 weeks; 3) following the interval 6-week washout period (i.e. at the start of the second 10-week period of the crossover treatment); and 4) at the end of the second 10-week period. We also measured 24-hour ambulatory blood pressure at each of the four time points [20]. The specific assays, methods, and equipment used to measure these MetS components have been described [15].

### Outcomes

The primary outcome was the change in each of the five MetS components during melatonin treatment compared with placebo. A secondary outcome was the proportion of subjects free from MetS following melatonin versus placebo treatment after the first 10-week period, and again after the second 10-week period. The incidence of major adverse clinical events and minor side effects were documented.

### Power calculations

This Phase II trial was designed to provide estimates of treatment effect size and variability [15]. Nonetheless, a target sample size of 30 patients completing the trial would result in statistical power being between 0.79 to 0.86 utilizing a two-sided Type I error rate alpha of 0.05 for the following mean within-subject differences (for melatonin minus placebo) for each of the following MetS components: fasting glucose, -5.5 mg/dL; systolic blood pressure, -5.5 mmHg; HDL cholesterol, +3.6 mg/dL; triglycerides, -7.5 mg/dL; and waist circumference, - 4.0 cm, with estimated standard deviations as previously reported [15]. We also calculated the statistical power for the “global” test of whether melatonin would improve at least one of the five MetS components, based on the multivariate Hotelling T^2^ in PASS 2005 (Kaysville, UT), with >90% power with a two-sided alpha of 0.05.

### Statistical analysis

Analysis of the primary endpoints was performed according to the intention-to-treat principle. Standard statistical methods for a one-period crossover design were employed using SAS® software (Cary, NC, USA), including testing for both a carryover effect and an order effect [21]. Only two subjects dropped out during follow-up, which was deemed unrelated to study drug. Continuous variables were summarized using means and standard deviations, and counts with percentages. Two-sided P values for the primary endpoint were calculated using a mixed model. We performed interaction tests to determine whether the effect of treatment differed by sex or race. Fisher’s exact test was used to analyze the proportion of subjects free from MetS following melatonin versus placebo after the first and second 10-week periods and to compare the incidence of reported adverse effects for melatonin versus placebo.

## Results

### Baseline characteristics

Baseline characteristics are shown in Table 1. Of the 39 randomized subjects, 19 received melatonin and 20 placebo in the first 10-week period. Although all 39 subjects had MetS at screening, at the first post-randomization study visit only 25 (64%) had at least three MetS components, whereas 14 (36%) had only 1 or 2 MetS components and, therefore, no longer had clinical MetS.

**Table 1:** Baseline characteristics according to the treatment which subjects were randomized to receive during the first 10-week period of the crossover trial*

Characteristic	Melatonin first	Placebo first
N	19	20
Age, years (mean, SD)	62.7 ± 9.6	57.6 ± 10.1
Sex (N, %)		
Male	9 (52.6%)	8 (40.0%)
Female	10 (47.4%)	12 (60.0%)
Race (N, %)		
White	13 (68.4%)	11 (55.0%)
African-American	6 (31.6%)	7 (35.0%)
Asian	0 (0.0%)	1 (5.0%)
Hispanic/Latino	0 (0.0%)	1 (5.0%)
Waist circumference, cm (mean, SD)		
Male	114.1 ± 11.1	110.4 ± 5.3
Female	102.6 ± 8.8	104.7 ± 11.4
Height, meters (mean, SD)	1.67 ± 0.11	1.70 ± 0.12
Weight, kg (mean, SD)	97.5 ± 19.4	98.1 ± 14.3
Body mass index (mean, SD)	35.2 ± 7.0	34.1 ± 6.4
HDL-cholesterol, mg/dL (mean, SD)		
Male	37.7 ± 6.7	35.7 ± 6.3
Female	44.7 ± 6.7	48.5 ± 12.5
LDL-cholesterol, mg/dL (mean, SD)	115.2 ± 33.6	115.9 ± 34.1
Triglycerides, mg/dL (mean, SD)	152.0 ± 59.3	210.1 ± 227.5
Fasting glucose, mg/dL (mean, SD)	102.2 ± 14.7	103.0 ± 13.3
SBP average clinic value, mmHg (mean, SD)	125.7 ± 14.5	126.5 ± 19.4
DBP average clinic value, mmHg (mean, SD)	76.5 ± 10.9	77.7 ± 12.0
Ambulatory blood pressure readings (mmHg)		
Full 24-hour period		
Measurements per subject (mean, SD)	22.7 ± 2.7	22.4 ± 2.6
Average SBP, mmHg (mean, SD)	126.4 ± 9.6	132.4 ± 9.3
Average DBP, mmHg (mean, SD)	74.2 ± 9.6	77.5 ± 9.0
Wake period (0800 to 2200)		
Measurements per subject (mean, SD)	13.4 ± 2.4	13.2 ± 1.8
Average SBP, mmHg (mean, SD)	131.3 ± 10.1	135.5 ± 9.8
Average DBP, mmHg (mean, SD)	78.3 ± 7.4	80.7 ± 9.3
Sleep period (2200 to 0800)		
Measurements per subject (mean, SD)	9.3 ± 1.0	9.2 ± 1.6
Average SBP, mmHg (mean, SD)	119.9 ± 9.7	127.5 ± 10.9
Average DBP, mmHg (mean, SD)	69.1 ± 7.1	72.5 ± 9.5
Subjects with each of the following MetS components (N, %)		
Waist circumference ≥102 cm (male) or ≥88 cm (female)	19 (100.0%)	19 (95.0%)
HDL cholesterol <40 mg/dL (male) or <50 mg/dL (female)	14 (73.7%)	17 (85.0%)
Triglycerides ≥150 mg/dL	11 (57.9%)	11 (55.0%)
Fasting glucose ≥100 mg/dL	14 (73.7%)	13 (65.0%)
SBP ≥130 mmHg or DBP ≥85 mmHg	12 (63.2%)	16 (80.0%)
Number of MetS components per subject (mean, SD)	3.68 ± 0.67	3.80 ± 0.95
Proportion of subjects with MetS at the time of randomization (N, %)	11 (57.9%)	14 (70.0%)
Proportion with different number of components of MetS (N, %)		
0	0 (0.0%)	0 (0.0%)
1	2 (10.5%)	1 (5.0%)
2	6 (31.6%)	5 (25.0%)
3	7 (36.8%)	10 (50.0%)
4	4 (21.1%)	2 (10.0%)
5	0 (0.0%)	2 (10.0%)

*P values are not presented comparing melatonin with placebo for baseline characteristics, because this is a crossover trial (and not a parallel group trial), in which all 39 subjects received both melatonin and placebo (or placebo and melatonin) in sequential order, and therefore each subject serves as his or her own control.

### Primary endpoint: change in each individual MetS component with treatment

**Figure 3:**
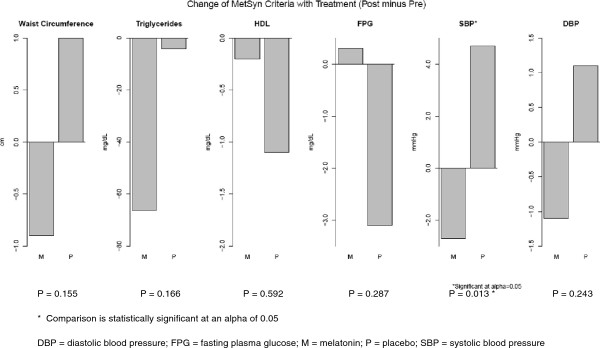
**Change in metabolic syndrome criteria with melatonin versus placebo treatment.** The change value for each treatment was calculated by subtracting the baseline value from the follow-up value at the end of the 10-week treatment period. Mixed model P values are presented (as described in the text).

Table 2 and Figure 3 present the change before and after melatonin versus placebo treatment for each MetS component. Melatonin was associated with a modest reduction in mean waist circumference (-0.9 cm) compared with a modest increase for placebo (+1.0 cm; p = 0.15). Melatonin was associated with a mean decrease of 66 mg/dL in triglycerides, compared with a 4 mg/dL decrease with placebo (p = 0.166). For HDL cholesterol, both melatonin and placebo were associated with very small reductions (-0.2 mg/dL and -1.1 mg/dL respectively, p = 0.59). For fasting glucose, melatonin was associated with a 0.3 mg/dL increase, compared with a 3.1 mg/dL reduction for placebo (p = 0.29). Mean clinic SBP dropped by 2.7 mmHg for melatonin, compared with a 4.7 mmHg increase for placebo (p = 0.013). Mean clinic DBP dropped by 1.1 mmHg for melatonin versus a 1.1 mmHg increase for placebo (p = 0.24). Ambulatory SBP dropped slightly for melatonin (-1.4 mmHg) and increased with placebo (+0.3 mmHg, p > 0.4), and ambulatory DBP dropped slightly for melatonin (-1.0 mmHg) and increased with placebo (+0.4 mmHg, p > 0.4). When ambulatory blood pressure readings were stratified into wake periods (0800 to 2200) and sleeping periods (2200 to 0800), melatonin reduced the wake average SBP by 2.2 mmHg (versus a 1.4 mmHg increase with placebo, p =0.169), and reduced the average DBP by 1.8 mmHg (versus a 1.8 mmHg increase with placebo, p = 0.044). In contrast, melatonin did not improve SBP or DBP compared with placebo during the sleeping periods (Table 2).

**Table 2:** Main results comparing the effect of melatonin with placebo on the components of the metabolic syndrome*

Parameter	Melatonin	Placebo	P value (mixed model)
Weight, kg (mean, SD)			
Sample size (pre, post)	(n = 39, 38)	(n = 38, 37)	
Pre	97.6 ± 16.9	96.2 ± 15.7	
Post	95.8 ± 15.9	96.8 ± 15.7	
Change with treatment (post minus pre)	-1.4 ± 4.4	-0.02 ± 1.8	0.087
BMI (mean, SD)			
Sample size (pre, post)	(n = 39, 38)	(n = 38, 37)	
Pre	34.6 ± 6.7	33.8 ± 6.0	
Post	33.7 ± 6.1	34.0 ± 6.0	
Change with treatment (post minus pre)	-0.4 ± 1.4	-0.0 ± 0.7	0.090
Waist circumference, cm (mean, SD)			
Sample size (pre, post)	(n = 37, 37)	(n = 38, 37)	
Pre	108.8 ± 12.2	106.8 ± 10.5	
Post	108.1 ± 11.3	108.0 ± 10.6	
Change with treatment (post minus pre) (n = 35 Melatonin)	-0.9 ± 5.9	1.0 ± 5.5	0.155
Triglycerides, mg/dL (mean, SD)			
Sample size (pre, post)	(n = 39, 38)	(n = 38, 37)	
Pre	207.2 ± 271.9	175.3 ± 172.1	
Post	142.6 ± 74.1	172.4 ± 216.9	
Change with treatment (post minus pre)	-66.3 ± 211.8	-4.2 ± 99.6	0.166
LDL cholesterol, mg/dL (mean, SD)			
Sample size (pre, post)	(n = 39, 38)	(n = 38, 37)	
Pre	112.6 ± 32.3	112.7 ± 31.2	
Post	108.5 ± 33.2	114.6 ± 35.3	
Change with treatment (post minus pre)	-3.7 ± 25.6	0.1 ± 25.5	0.564
HDL cholesterol, mg/dL (mean, SD)			
Sample size (pre, post)	(n = 39, 38)	(n = 38, 37)	
Pre	40.8 ± 7.6	42.5 ± 10.1	
Post	40.9 ± 8.3	41.5 ± 8.3	
Change with treatment (post minus pre)	-0.2 ± 5.8	-1.1 ± 8.2	0.592
Fasting glucose, mg/dL (mean, SD)			
Sample size (pre, post)	(n = 39, 37)	(n = 38, 37)	
Pre	103.4 ± 16.5	104.6 ± 13.7	
Post	102.9 ± 17.1	101.9 ± 10.2	
Change with treatment (post minus pre)	0.3 ± 13.6	-3.1 ± 11.0	0.287
SBP average clinic value, mmHg (mean, SD)			
Sample size (pre, post)	(n = 39, 38)	(n = 38, 37)	
Pre	126.8 ± 16.3	122.3 ± 17.3	
Post	123.4 ± 15.2	127.0 ± 15.1	
Change with treatment (post minus pre)	-2.7 ± 11.3	4.7 ± 13.8	0.013
DBP average clinic value, mmHg (mean, SD)			
Sample size (pre, post)	(n = 39, 38)	(n = 38, 37)	
Pre	76.6 ± 11.3	75.3 ± 10.3	
Post	75.0 ± 10.8	76.4 ± 10.0	
Change with treatment (post minus pre)	-1.1 ± 6.9	1.1 ± 7.4	0.243
**Ambulatory BP readings**			
Full 24-hour period			
Average SBP, mmHg (mean, SD)			
Sample size (pre, post)	(n = 37, 38)	(n = 38, 37)	
Pre	131.6 ± 11.6	130.4 ± 9.5	
Post	129.6 ± 12.0	130.9 ± 12.0	
Change with treatment (post minus pre) (n = 36 Melatonin)	-1.4 ± 8.1	0.3 ± 8.5	0.475
Average DBP, mmHg (mean, SD)			
Sample size (pre, post)	(n = 37, 38)	(n = 38, 37)	
Pre	76.9 ± 9.0	76.3 ± 8.2	
Post	75.7 ± 8.6	76.8 ± 8.3	
Change with treatment (post minus pre) (n = 36 Melatonin)	-1.0 ± 6.3	0.4 ± 5.7	0.409
Wake period (0800 to 2200)			
Average SBP, mmHg (mean, SD)			
Sample size (pre, post)	(n = 37, 38)	(n = 38, 37)	
Pre	136.1 ± 12.4	134.2 ± 9.3	
Post	133.2 ± 12.2	135.8 ± 12.6	
Change with treatment (post minus pre) (n = 36 Melatonin)	-2.2 ± 9.1	1.4 ± 10.5	0.169
Average DBP, mmHg (mean, SD)			
Sample size (pre, post)	(n = 37, 38)	(n = 38, 37)	
Pre	81.0 ± 9.6	79.8 ± 8.0	
Post	78.9 ± 9.1	81.6 ± 8.7	
Change with treatment (post minus pre) (n = 36 Melatonin)	-1.8 ± 7.3	1.8 ± 6.6	0.044
Sleep period (2200 to 0800)			
Average SBP, mmHg (mean, SD)			
Sample size (pre, post)	(n = 37, 36)	(n = 38, 37)	
Pre	125.8 ± 12.3	125.1 ± 11.5	
Post	124.9 ± 13.4	123.9 ± 13.5	
Change with treatment (post minus pre) (n = 34 Melatonin)	-1.1 ± 9.0	-1.4 ± 8.9	0.807
Average DBP, mmHg (mean, SD)			
Sample size (pre, post)	(n = 37, 36)	(n = 38, 37)	
Pre	71.7 ± 9.1	71.2 ± 9.5	
Post	70.7 ± 8.8	69.9 ± 8.7	
Change with treatment (post minus pre) (n = 34 Melatonin)	-0.7 ± 6.5	-1.3 ± 6.7	0.729

*The mean values in the “Change with treatment (post minus pre)” rows may not equal exactly the difference between the above corresponding “pre” and “post” rows because of different sample sizes due to subject drop out in the post- versus the pre-period.

DBP = diastolic blood pressure; SBP = systolic blood pressure; SD = standard deviation.

### Secondary endpoint: freedom from MetS after study treatment

Of the 39 randomized subjects, 37 completed measurements of all five MetS components at all four study measurement periods (before and after the first and second 10-week periods). After the first 10-week period, 6 of 17 (35.3%) of subjects taking melatonin compared with 3 of 20 (15.0%) of those taking placebo were free from MetS (p = 0.25). After the second 10-week period, 9 of 20 (45.0%) taking melatonin compared with 4 of 17 (23.5%) taking placebo were free from MetS (p = 0.30).

### Adverse effects, clinical events, and adherence

There were no differences in the reported incidence of these side effects between treatments (Table 3). One subject experienced an acute stroke 10 days after being randomized to melatonin, which was determined to be unlikely due to study drug. The subject completed the study without any other major vascular events. The proportions of subjects adherent to study drug (defined as taking at least 80% of dispensed tablets) was 29 of 33 (87.9%) for melatonin, compared with 25 of 34 (73.5%) for placebo (p = 0.22). Adherence for study drug tended to decline from the first 10-week period (30 of 34, or 88.2% adherent) to the second 10-week period (24 of 33, or 72.7% adherent, p = 0.13).

**Table 3:** **Safety, tolerability, and adverse effects of melatonin and placebo**

Parameter	Melatonin	Placebo	Fisher’s P value
N	39	39	
Nausea, patients reporting "Yes" (N, %)			
Pre	2/39 (5.1%)	3/38 (7.9%)	0.675
Post	3/38 (7.9%)	4/36 (11.1%)	0.707
Dizziness, patients reporting "Yes" (N, %)			
Pre	8/39 (20.5%)	8/38 (21.1%)	1.00
Post	8/38 (21.1%)	10/35 (28.6%)	0.588
Fatigue, patients reporting "Yes" (N, %)			
Pre	20/39 (51.3%)	15/38 (39.5%)	0.363
Post	23/38 (60.5%)	19/36 (52.8%)	0.639
Drowsiness, patients reporting "Yes" (N, %)			
Pre	22/39 (56.4%)	18/37 (48.6%)	0.646
Post	22/37 (59.5%)	17/36 (47.2%)	0.352
Early morning awakening or sleep disturbances, patients reporting "Yes" (N, %)			
Pre	19/39 (48.7%)	22/37 (59.5%)	0.368
Post	22/37 (59.5%)	23/36 (63.9%)	0.811

### Blinding, carry over effect, order effect

Patients were able to guess correctly more often than incorrectly which treatment (melatonin or placebo) they were randomized to receive first. Of 37 subjects completing the final visit, 20 (54.1%) correctly guessed their treatment assignment, 8 (21.6%) guessed incorrectly, and 9 (24.3%) documented not knowing (p = 0.053). For each of the five MetS components, there was no detectable carryover effect (p >0.25 in all cases), and no order effect (p >0.05 in all cases).

### Differences by sex and race

There were no significant interactions by sex (all p values >0.10). For race, however, interaction P values for clinic SBP (p =0.025) and clinic DBP (p =0.045) were statistically significant. Accordingly, we stratified the results by race (Table 4). Among white participants, mean SBP and DBP fell modestly with melatonin (-2.6 mmHg and -1.3 mmHg respectively), and mean SBP increased with placebo (+1.7 mmHg) with no change in DBP. Among African-Americans, mean SBP and DBP also fell modestly with melatonin (-3.9 mmHg and -1.4 mmHg respectively), but there was a greater increase in SBP and DBP with placebo (+12.5 mmHg and +4.9 mmHg respectively).

**Table 4:** **Results of melatonin versus placebo on clinic blood pressure, stratified by African-American or white race**

Parameter	Melatonin	Placebo	P value (mixed model)
**SBP average clinic value, mmHg (mean, SD)**	(n = 39, 38)	(n = 38, 37)	
Whites (n = 22)			
Pre	123.2 ± 12.7	121.4 ± 11.4	
Post	119.4 ± 10.9	122.9 ± 12.4	
Change with treatment (post minus pre)	-2.6 ± 11.0	1.7 ± 12.0	0.196
African-Americans (n = 13)			
Pre	131.0 ± 21.0	119.1 ± 22.1	
Post	127.1 ± 18.4	131.6 ± 18.0	
Change with treatment (post minus pre)	-3.9 ± 10.9	12.5 ± 13.7	0.003
**DBP average clinic value, mmHg (mean, SD)**	(n = 39, 38)	(n = 38, 37)	
Whites (n = 22)			
Pre	73.3 ± 8.4	73.1 ± 5.9	
Post	71.1 ± 5.6	72.9 ± 6.3	
Change with treatment (post minus pre)	-1.3 ± 7.2	0.0 ± 5.0	0.840
African-Americans (n = 13)			
Pre	80.7 ± 14.2	76.2 ± 13.7	
Post	79.3 ± 14.4	81.1 ± 13.3	
Change with treatment (post minus pre)	-1.4 ± 6.6	4.9 ± 8.6	0.034

DBP = diastolic blood pressure; SBP = systolic blood pressure; SD = standard deviation.

## Discussion

In this Phase II crossover trial, treatment with melatonin compared with placebo was associated with modest improvements in most individual MetS components, as well as a tendency towards more subjects being free from MetS. Tolerability and adherence were comparable between melatonin and placebo throughout the study. We found no evidence of any order effect or carryover effect, suggesting that the crossover design and the order of study treatments did not discernibly bias our results.

Prior trials have been conducted to assess the impact of melatonin on one or more MetS factors. Most of these studies evaluated the effect of melatonin on blood pressure, and demonstrated a reduction in blood pressure associated with melatonin treatment [22–29]. Our trial similarly demonstrated an improvement in SBP associated with melatonin versus placebo. Based on 24-hour ambulatory blood pressure data, the change in blood pressure following melatonin versus placebo treatment was more pronounced during the awake period (0800 to 2200) compared with the sleeping period (2200 to 0800). However, another trial reported that melatonin lowered blood pressure at night but raised it during the daytime compared with placebo [30]. Our trial also found that melatonin was associated with a modest improvement in HDL cholesterol and triglyceride levels. Prior melatonin trials have shown conflicting results, with some reporting improved lipid profiles [31, 32]; some reporting worsened triglycerides [33, 34]; and others reporting no effect [35, 36]. Our study found melatonin to be associated with a slight, non-significant worsening in fasting plasma glucose compared with placebo. Our findings contrast those of prior genome-wide association studies implicating the melatonin signaling pathway and glucose homeostasis [8–10], as well as epidemiologic studies that suggest an inverse relationship between nocturnal melatonin secretion and insulin resistance [13, 14]. However, similar to our study, other trials have also shown that supplemental melatonin may impair glucose tolerance [37], or have a neutral effect [35]. These discrepancies may be due, at least in part, to differences in the tested doses of melatonin (ranging from 0.3 to 10.0 mg daily in prior studies) and/or in the use of controlled-release versus fast-release melatonin [38], and suggest that relationships between supplemental melatonin, measures of endogenous melatonin levels, and MetS parameters are complex.

Unlike prior studies, our trial prospectively measured all five MetS components before and after melatonin and placebo treatment, instead of focusing on only one or two MetS components. This is a strength of our trial, given that the metabolic risk factors that comprise MetS tend to cluster. Our trial showed that melatonin tended to increase the proportion of subjects free from MetS compared with placebo. Even though our study was not powered to provide definitive conclusions for this endpoint, it suggests that melatonin’s modest benefits on multiple individual MetS components may be additive and sufficient enough to reverse the diagnosis of MetS in some cases.

In our study, there was a race interaction for clinic SBP and DBP, such that melatonin produced appreciably greater improvements in SBP and DBP in African-American participants. We consider these findings in light of prior reports that shift work is associated with hypertension more often in African-Americans than in whites [39], and that African-Americans may have more blunted circadian declines in nocturnal blood pressure [40, 41]. These racial differences in circadian blood pressure variability provide a plausible explanation for melatonin’s greater efficacy in African-Americans compared with white participants in our trial.

Our trial has several limitations. First, it was designed as a pilot study to determine the effect of melatonin compared with placebo on individual MetS components; it was not powered to determine whether melatonin could increase the proportion of subjects free from MetS following melatonin versus placebo (even though we reported this as a secondary endpoint). Second, the crossover study design enabled each subject to serve as his or her own control and provided greater statistical power for a given sample size. However, because each subject received both melatonin and placebo at different periods, subjects were able to guess which study treatment they were randomized to receive first more often than expected by chance, potentially compromising blinding to study drug. It is possible that changes in sleep patterns or sides effects of melatonin were responsible for being able to guess treatment received, even though there were no differences in the reported rate of sleep disturbances or other adverse effects during the trial (Table 3). It is unlikely that participants’ suspicion regarding which therapy they received first affected the primary endpoint (i.e. objective measures of MetS parameters), particularly since we demonstrated no carryover or order effect in the trial. Third, separate measurements for MetS criteria at study screening and again following randomization were required to ensure a comparable assessment of study drug efficacy during the first and second 10-week period in this crossover trial, which resulted in 14 of 39 patients (36%) no longer having three or more MetS components after randomization. This was likely due to regression to the mean of individual MetS components.

## Conclusions

In this Phase II crossover trial of subjects with MetS, oral melatonin produced modest improvements in most MetS components, and also tended to increase the proportion of subjects free from MetS compared with placebo. Melatonin was well-tolerated and adherence to study drug was high. These findings support the conduct of a subsequent, larger, parallel-group trial of MetS subjects to determine whether melatonin is more efficacious than placebo at treating MetS, as well as its downstream cardiovascular and metabolic complications.

## Abbreviations

DBP: Diastolic blood pressure
MetS: Metabolic syndrome
SBP: Systolic blood pressure.

## References

[1] Mozumdar A, Liguori G (2011). Persistent increase of prevalence of metabolic syndrome among U.S. adults: NHANES III to NHANES 1999–2006. Diabetes Care.

[2] Ford ES (2005). Risks for all-cause mortality, cardiovascular disease, and diabetes associated with the metabolic syndrome: a summary of the evidence. Diabetes Care.

[3] Pevet P, Challet E (2011). Melatonin: both master clock output and internal time-giver in the circadian clocks network. J Physiology-Paris.

[4] Peschke E, Bach AG, Mühlbauer E (2006). Parallel signaling pathways of melatonin in the pancreatic β-cell. J Pineal Res.

[5] Ríos-Lugo MJ, Cano P, Jiménez-Ortega V, Fernández-Mateos MP, Scacchi PA, Cardinali DP, Esquifino AI (2010). Melatonin effect on plasma adiponectin, leptin, insulin, glucose, triglycerides and cholesterol in normal and high fat–fed rats. J Pineal Res.

[6] Nduhirabandi F, du Toit EF, Lochner A (2012). Melatonin and the metabolic syndrome: a tool for effective therapy in obesity-associated abnormalities?. Acta Physiol.

[7] Koziróg M, Poliwczak AR, Duchnowicz P, Koter-Michalak M, Sikora J, Broncel M (2011). Melatonin treatment improves blood pressure, lipid profile, and parameters of oxidative stress in patients with metabolic syndrome. J Pineal Res.

[8] Bouatia-Naji N, Bonnefond A, Cavalcanti-Proenca C, Sparso T, Holmkvist J, Marchand M, Delplanque J, Lobbens S, Rocheleau G, Durand E, De Graeve F, Chèvre JC, Borch-Johnsen K, Hartikainen AL, Ruokonen A, Tichet J, Marre M, Weill J, Heude B, Tauber M, Lemaire K, Schuit F, Elliott P, Jørgensen T, Charpentier G, Hadjadj S, Cauchi S, Vaxillaire M, Sladek R, Visvikis-Siest S (2009). A variant near MTNR1B is associated with increased fasting plasma glucose levels and type 2 diabetes risk. Nat Genet.

[9] Prokopenko I, Langenberg C, Florez JC, Saxena R, Soranzo N, Thorleifsson G, Loos RJF, Manning AK, Jackson AU, Aulchenko Y, Potter SC, Erdos MR, Sanna S, Hottenga JJ, Wheeler E, Kaakinen M, Lyssenko V, Chen WM, Ahmadi K, Beckmann JS, Bergman RN, Bochud M, Bonnycastle LL, Buchanan TA, Cao A, Cervino A, Coin L, Collins FS, Crisponi L, de Geus EJ (2009). Variants in MTNR1B influence fasting glucose levels. Nat Genet.

[10] Bonnefond A, Clement N, Fawcett K, Yengo L, Vaillant E, Guillaume J-L, Dechaume A, Payne F, Roussel R, Czernichow S, Hercberg S, Hadjadj S, Balkau B, Marre M, Lantieri O, Langenberg C, Bauatia-Naji N, Charpentier G, Vaxillaire M, Rocheleau G, Wareham NJ, Sladek R, McCarthy MI, Dina C, Barroso I, Jockers R, Froguel P, Meta-Analysis of Glucose and Insulin-Related Traits Consurtium (MAGIC) (2012). Rare MTNR1B variants impairing melatonin receptor 1B function contribute to type 2 diabetes. Nat Genet.

[11] Mäntele S, Otway DT, Middleton B, Bretschneider S, Wright J, Robertson MD, Skene DJ, Johnston JD (2012). Daily rhythms of plasma melatonin, but not plasma leptin or leptin mRNA, vary between lean, obese and type 2 diabetic men. PLoS ONE.

[12] Corbalán-Tutau D, Madrid JA, Nicolás F, Garaulet M (2014). Daily profile in two circadian markers “melatonin and cortisol” and associations with metabolic syndrome components. Physiol Behav.

[13] McMullan CJ, Curhan GC, Schernhammer ES, Forman JP (2013). Association of nocturnal melatonin secretion with insulin resistance in nondiabetic young women. Am J Epidemiol.

[14] McMullan CJ, Schernhammer ES, Rimm EB, Hu FB, Forman JP (2013). Melatonin secretion and the incidence of type 2 diabetes. JAMA.

[15] Terry PD, Goyal A, Phillips LS, Superak HM, Kutner MH (2013). Design and rationale of a randomized controlled trial of melatonin supplementation in men and women with the metabolic syndrome. Open Access J Clin Trials.

[16] CONsolidated Standards of Reporting Trials (CONSORT) checklistDownloaded from . Accessed on October 13, 2013 http://www.consort-statement.org

[17] Grundy SM, Brewer JHB, Cleeman JI, Smith JSC, Lenfant C (2004). Definition of the metabolic syndrome: report of the National Heart, Lung, and Blood Institute/American Heart Association conference on scientific issues related to the definition. Circulation.

[18] Lusardi P, Piazza E, Fogari R (2000). Cardiovascular effects of melatonin in hypertensive patients well controlled by nifedipine: a 24-hour study. Br J Clin Pharmacol.

[19] Buscemi N, Vandermeer B, Hooton N, Pandya R, Tjosvold L, Hartling L, Vohra S, Klassen TP, Baker G (2006). Efficacy and safety of exogenous melatonin for secondary sleep disorders and sleep disorders accompanying sleep restriction: meta-analysis. BMJ.

[20] Baumgart P, Kamp J (1998). Accuracy of the SpaceLabs Medical 90217 ambulatory blood pressure monitor. Blood Press Monit.

[21] Altman DG (1991). Practical Statistics for Medical Research.

[22] Arangino S, Cagnacci A, Angiolucci M, Vacca A, Longu G, Volpe A, Melis GB (1999). Effects of melatonin on vascular reactivity, catecholamine levels, and blood pressure in healthy men. Am J Cardiol.

[23] Nishiyama K, Yasue H, Moriyama Y, Tsunoda R, Ogawa H, Yoshimura M, Kugiyama K (2001). Acute effects of melatonin administration on cardiovascular autonomic regulation in healthy men. Am Heart J.

[24] Scheer FA, Van Montfrans GA, van Someren EJ, Mairuhu G, Buijs RM (2004). Daily nighttime melatonin reduces blood pressure in male patients with essential hypertension. Hypertension.

[25] Grossman E, Laudon M, Yalcin R, Zengil H, Peleg E, Sharabi Y, Kamari Y, Shen-Orr Z, Zisapel N (2006). Melatonin reduces night blood pressure in patients with nocturnal hypertension. Am J Med.

[26] Cagnacci A, Arangino S, Angiolucci M, Maschio E, Melis GB (1998). Influences of melatonin administration on the circulation of women. Am J Physiol-Reg I.

[27] Cagnacci A, Arangino S, Angiolucci M, Melis GB, Facchinetti F, Malmusi S, Volpe A (2001). Effect of exogenous melatonin on vascular reactivity and nitric oxide in postmenopausal women: role of hormone replacement therapy. Clin Endocrinol.

[28] Cagnacci A, Cannoletta M, Renzi A, Baldassari F, Arangino S, Volpe A (2005). Prolonged melatonin administration decreases nocturnal blood pressure in women. Am J Hypertens.

[29] Lusardi P, Preti P, Savino S, Piazza E, Zoppi A, Fogari R (1997). Effect of bedtime melatonin ingestion on blood pressure of normotensive subjects. Blood Press Monit.

[30] Rechcinski T, Trzos E, Wierzbowska-Drabik K, Krzeminska-Pakula M, Kurpesa M (2009). Melatonin for nondippers with coronary artery disease: assessment of blood pressure profile and heart rate variability. Hypertens Res.

[31] Pittalis S, Lissoni P, Giani L, Casati M, Kropacek N, Orfanò S, Giavolucci F, Merlini D (1997). Effect of a chronic therapy with the pineal hormone melatonin on cholesterol levels in idiopathic hypercholesterolemic patients. Recent Progr Med.

[32] Tamura H, Nakamura Y, Narimatsu A, Yamagata Y, Takasaki A, Reiter RJ, Sugino N (2008). Melatonin treatment in peri‒and postmenopausal women elevates serum high‒density lipoprotein cholesterol levels without influencing total cholesterol levels. J Pineal Res.

[33] Wakatsuki A, Okatani Y, Ikenoue N, Izumiya C, Kaneda C (2000). Melatonin inhibits oxidative modification of low‒density lipoprotein particles in normolipidemic post‒menopausal women. J Pineal Res.

[34] Wakatsuki A, Okatani Y, Ikenoue N, Kaneda C, Fukaya T (2001). Effects of short-term melatonin administration on lipoprotein metabolism in normolipidemic postmenopausal women. Maturitas.

[35] Pawlikowski M, Kolomecka M, Wojtczak A, Karasek M (2002). Effects of six months melatonin treatment on sleep quality and serum concentrations of estradiol, cortisol, dehydroepiandrosterone sulfate, and somatomedin C in elderly women. Neuroendocrinol Lett.

[36] Rindone JP, Achacoso R (1997). Effect of melatonin on serum lipids in patients with hypercholesterolemia: a pilot study. Am J Ther.

[37] Cagnacci A, Arangino S, Renzi A, Paoletti AM, Melis GB, Cagnacci P, Volpe A (2001). Influence of melatonin administration on glucose tolerance and insulin sensitivity of postmenopausal women. Clin Endocrinol.

[38] Grossman E, Laudon M, Zisapel N (2011). Effect of melatonin on nocturnal blood pressure: meta-analysis of randomized controlled trials. Vasc Health Risk Manag.

[39] Lieu SJ, Curhan GC, Schernhammer ES, Forman JP (2012). Rotating night shift work and disparate hypertension risk in African–Americans. J Hypertens.

[40] Jehn ML, Brotman DJ, Appel LJ (2008). Racial differences in diurnal blood pressure and heart rate patterns: results from the Dietary Approaches to Stop Hypertension (DASH) trial. Arch Internal Med.

[41] Profant J, Dimsdale JE (1999). Race and diurnal blood pressure patterns: a review and meta-analysis. Hypertension.

